# Extracellular Matrix and the Production of Cultured Meat

**DOI:** 10.3390/foods10123116

**Published:** 2021-12-15

**Authors:** Khurshid Ahmad, Jeong-Ho Lim, Eun-Ju Lee, Hee-Jin Chun, Shahid Ali, Syed Sayeed Ahmad, Sibhghatulla Shaikh, Inho Choi

**Affiliations:** 1 Department of Medical Biotechnology, Yeungnam University, Gyeongsan 38541, Korea; ahmadkhursheed2008@gmail.com (K.A.); lim2249@naver.com (J.-H.L.); gorapadoc0315@hanmail.net (E.-J.L.); po98053@gmail.com (H.-J.C.); ali.ali.md111@gmail.com (S.A.); sayeedahmad4@gmail.com (S.S.A.); sibhghat.88@gmail.com (S.S.); 2 Research Institute of Cell Culture, Yeungnam University, Gyeongsan 38541, Korea

**Keywords:** cultured meat, skeletal muscle, extracellular matrix, scaffold

## Abstract

Cultured meat production is an evolving method of producing animal meat using tissue engineering techniques. Cells, chemical factors, and suitable biomaterials that serve as scaffolds are all essential for the cultivation of muscle tissue. Scaffolding is essential for the development of organized meat products resembling steaks because it provides the mechanical stability needed by cells to attach, differentiate, and mature. In in vivo settings, extracellular matrix (ECM) ensures substrates and scaffolds are provided for cells. The ECM of skeletal muscle (SM) maintains tissue elasticity, creates adhesion points for cells, provides a three-dimensional (3D) environment, and regulates biological processes. Consequently, creating mimics of native ECM is a difficult task. Animal-derived polymers like collagen are often regarded as the gold standard for producing scaffolds with ECM-like properties. Animal-free scaffolds are being investigated as a potential source of stable, chemically defined, low-cost materials for cultured meat production. In this review, we explore the influence of ECM on myogenesis and its role as a scaffold and vital component to improve the efficacy of the culture media used to produce cultured meat.

## 1. Introduction

Demographic developments, economic gain, and urbanization have all contributed to an increase in global meat consumption and production. This growing demand is troublesome because modern large-scale animal husbandry practices have associated public health and environmental pollution problems and raise questions regarding animal welfare [1]. Foodborne infections, diet-associated diseases, infectious diseases, and antibiotic resistance are also linked to the animal agricultural sector [2]. Furthermore, meat consumption in developing countries is anticipated to increase due to high population levels and growth rates, and these increases are predicted to result in a five-fold greater increase in the amount of meat consumed in developing countries as compared with developed countries [3].

Cultured meat, which is also called artificial meat, lab-grown meat, clean meat, cell-based meat, or in vitro meat, is a synthetic meat created in laboratories by cultivating muscle stem cells. Research on this topic was preceded by decades of experience in the cell culture, tissue engineering, stem cell biology, bioprocess, and chemical engineering fields [4]. Instead of being sourced directly from slaughtered animals, cultured meat is composed of animal cells and is produced using a variety of tissue engineering methods [5]. Year after year, the number of cultured meat production startups grows, for example, it increased from four or five in 2016 to 60 in June 2020 [6], and several companies are actively planning to produce cultured meat products in the near future [1].

Cells, media, and scaffolds are the three main raw materials required for cultured meat production, which consists of the following stages: (1) the isolation of stem cells from animals, (2) their submersion in a culture medium that provides all of the minerals, carbohydrates, amino acids, and fats the cells need to grow, proliferate, and differentiate, and (3) the transfer of these cells to a bioreactor, which exercises the cells and exposes them to growth factors that allow them to differentiate into muscle cells.

The majority of cultured meat production techniques focus primarily on the in vitro production and differentiation of muscle satellite (stem) cells (MSCs), which was first described in the first report issued on the cultivated muscle-based beef burgers [7]. MSCs are multipotent progenitor cells with a high potential for self-renewal and provide functional stability and preserve skeletal muscle (SM) integrity. The myogenic program, which is regulated by transcription factors, drives the proliferation and differentiation of MSCs into myotubes, that is, multinucleated muscle fibers produced during the developmental stage by the fusion of precursor myoblasts [8,9].

During the production of cultured meat, scaffold architecture is critical, and the ideal scaffold must offer a suitable 3D environment for cells to adhere, proliferate, and differentiate, while maintaining optimal porosity to enable the supply of nutrients and oxygen [10]. Tissues invariably contain ECM protein scaffolding that facilitates cell anchoring and tissue assembly via various membrane receptor proteins like integrins [11]. Collagen is one of the most abundant ECM proteins in SM, and accounts for 1–10% of the dry weight of muscle [12]. Collagen and collagen-derived gelatins are often utilized in the food and pharmaceutical industries due to their biocompatibilities, biodegradabilities, and low antigenicities. Collagens are widely regarded as the benchmark by those creating ECM-like scaffolds. Nonetheless, they are expensive because they are derived primarily from animals, and, aside from being expensive, it is not practical to use animal ingredients for cultured meat production, since this would undermine the purpose of producing meat in this way [13].

Aside from acting as a scaffold/backbone for cells, ECM is crucially required to improve the quality and optimize culture media. Several ECM proteins are used as supplements to increase the efficacy of growth and culture media. Notable attempts have been made to minimize the costs of media supplements and growth factors by replacing them with self-derived short peptides (e.g. FNINs, short peptides derived from fibronectin) [14] and using serum-free growth media and media components not obtained from animals. Progress is also being made to develop animal-free scaffolds, for example, from plant-derived biomaterials, that improve cell proliferation, differentiation, and tissue formation.

In this review, we discuss the role of ECM in myogenesis and ways of improving the efficiency of the media used for cultured meat production. Also, we review the use of ECM components as scaffolding materials for cultured meat production with a focus on non-animal derived, biodegradable, and edible cost-effective biomaterials.

## 2. Skeletal Muscle Myogenesis and Its Regulation

Essentially, edible animal meat is primarily comprised of SM tissues, and thus SM tissue engineering technologies are being utilized to produce cultured meat in vitro. Because adult SM cells are incapable of proliferating, MSCs are the only replication-competent precursors. Due to their high responsiveness and migratory abilities, MSCs are critical for preserving the structural and functional integrities of SM and are also responsible for muscle regeneration through a coordinated myogenic program [15]. MSCs are located between basal lamina and sarcolemma and actively regulate myofiber growth and progression under the influence of myogenic regulatory factors [12,16]. The discovery of MSCs enabled the production of cells in vitro and the development of cultured meat. Furthermore, MSCs provide a viable source of cells for SM recovery in vivo, and their ability to self-renew sustains stem cell populations and the generation of vast numbers of myogenic cells, which proliferate, multiply and fuse to form new myofibers [16]. The first cultured/clean meat production model was made using bovine MSCs, and this model was subsequently further developed in bioreactors for cultured meat production [17].

Myogenesis is a complex, highly ordered process regulated by the co-expressions of paired box transcription factors (Pax3/Pax7) and myogenic-regulatory factors such as Myf5, Mrf4, MyoD, and myogenin in MSCs [18,19,20]. Myogenesis is characterized by cell cycle arrest, myogenic activation, cell alignment, multiple cell fusion, an increase in nuclear sizes, and a peripheral localization [21], and SM regeneration is largely dependent on interactions between MSCs and their microenvironments (basal lamina and sarcolemma) [22].

Almost a decade ago, we began work on bovine primary cells, which provide an environment similar to that found in vivo, to explore the functions of genes involved in muscle development. Microarray analysis was performed to determine the functional significances of specific genes, particularly in terms of their differential expressions in the myogenic program [23,24]. We discovered that a number of ECM genes, such as FMOD (fibromodulin) [15,25], and MGP (matrix gla protein) [26] were significantly upregulated during myogenesis although, at the time, their roles in muscle development were unclear.

## 3. Skeletal Muscle Extracellular Matrix

ECM is a multifaceted environment that contains numerous molecules that provide structural support, cellular communications, and signal responses to injuries, and is considered to be responsible for the architectural conservation of SMs. ECM adheres to, interacts with, and protects muscle cells, facilitates biochemical signaling, provides structural support, and is essential during myogenesis [15], and many ECM proteins participate in cell-matrix interactions and matrix assembly regulation [27]. In addition to its biological functions, ECM includes nutrients such as proteins (mainly collagen) and glycosaminoglycans that influence tissue texture and overall meat quality [28].

ECM is also essential for the support and developmental regulation of myotubes during the early stages of myogenic differentiation [29] when ECM proteins interact with and regulate the functions and phenotypes of MSCs. Collagen fibers and proteoglycan matrix compose much of the ECM, but it also contains glycosaminoglycans and fibrous proteins such as elastin, fibronectin, and laminins [30]. A complex 3D ECM network occupies the basal lamina and is directly linked to MSCs [22]. 3D scaffolds are required to stabilize cells and imitate the ECM during tissue formation. To maximize the quality, taste, and tenderness of cultured meat, it may be beneficial to co-culture preadipocytes with myoblasts, because this effectively increases the intramuscular fat content of cultured meat.

**Figure 1:**
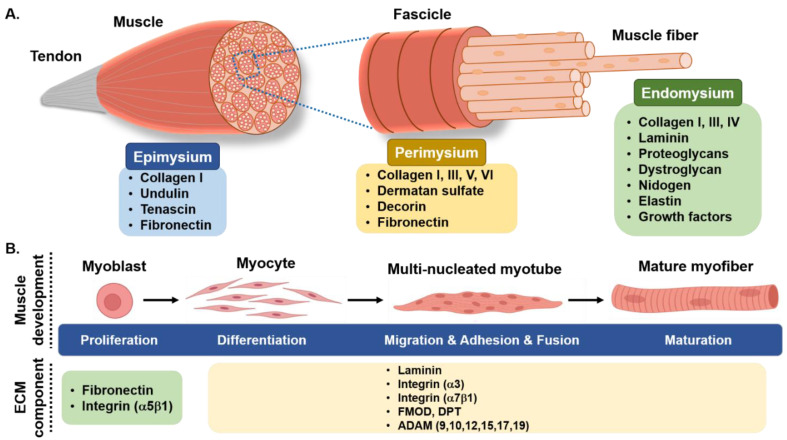
Skeletal muscle ECM: (**A**) three-layer structural schematic and associated ECM components; (**B**) Schematic representation of ECM-related protein activities during the different phases of myogenesis.

ECM functions as a scaffold for cell-matrix interactions, which are essential for a variety of physiological functions in muscle tissues. Collagens, laminin, and fibronectin are structural glycoproteins bound to proteoglycans that help preserve SM integrity and provide constructional support (Figure 1) [12]. Furthermore, several ECM proteins have been shown to control myogenesis. We previously examined a number of these proteins, such as fibromodulin (FMOD) [15,25], MGP (matrix gla protein) [26], and dermatopontin (DPT) [31], which play crucial roles in myogenesis regulation (Table 1). FMOD is a proteoglycan involved in ECM assembly and is closely associated with myogenesis [25]. On the other hand, DPT is a non-collagenous ECM constituent that is required for myogenesis regulation by increasing cell adhesion, decreasing proliferation, and enhancing differentiation [31]. DPT has also been shown to enhance the expressions of ECM-related genes (COL6A3, TNMD, MMP9, ELN) and inflammatory factors (TNF, IL6, IL8) in human visceral adipocytes [32], which suggests it participates in the regulation of ECM remodeling (Figure 1).

### 3.1. Collagen

Collagen is the most abundant fibrous protein in SM ECM and accounts for up to 10% of SM by weight, and, as mentioned above, collagen forms an intramuscular connective tissue network [27]. Collagens regulate cell attachment and differentiation and are largely responsible for the elasticity, tensile strength, and strength of bones [12]. Collagens are crucial for MSC self-renewal and differentiation in vivo, for example, the absence/knockout of type IV collagen impaired MSC regeneration capacity following muscle injury in mice [33]. Collagens come in a variety of forms, and several have been discovered in SM (Table 1) [12,34], such as the fibrillar collagens I, III, V, IX, and XI, and collagens I and III that account for more than 75% of total SM collagen [35].

Collagen and gelatins are extensively used in the food and pharmaceutical industries because of their biocompatibilities, biodegradabilities, and low antigenicities [36], and are also used to enhance cell adherence to suspended microcarriers and the microcarrier facilitated bead-to-bead translocation of bovine myoblasts. Microcarriers do not replicate the fibrous architecture of real muscle, and typically cells must be separated from microcarriers post-culture, which hinders culture and harvesting processes. While fibrillar structures may be replicated using fibrous gelatin, their scalability for food manufacturing purposes is limited by electrospinning or phase separation rates [37].

**Table 1:** ECM components and related proteins in skeletal muscle.

Protein Name		Function in Skeletal Muscle	Reference
Collagen	collagen I	collagen I, III, V, and XI form collagen fiber in the SM. collagen I promotes myoblast proliferation and migration while inhibiting myogenic differentiation	[27,29,38]
	collagen III		
	collagen V		
	collagen XI		
	collagen IV	promotes the regeneration of SM	
	collagen VI	maintains the physiological function of SM	
	collagen XII and XIV	localized primarily to perimysium and connect fibrillar collagen to other ECM constituents	
	collagen XV and XVIII	both collagen XV and XVIII bind growth factors	
		collagen XV provides mechanical support between cells and the ECM in SM fibers and microvessels.	
		have the ability to bind growth factors and contribute to the interaction of the basement membrane (BM) to other BM glycoproteins and endomysium	
Proteoglycans	decorin	regulator of type I collagen fibrillogenesis in SM	[27]
	perlecan	found in the BM and has a transient increase in expression throughout muscle differentiation	[39]
	fibromodulin	regulator of myostatin during myoblast differentiation	[15,25,40,41]
		regulates collagen fibrillogenesis	
	glycosaminoglycan	promotes myoblast proliferation and differentiation	[42]
	biglycan	binds with α- and γ-sarcoglycan of the dystrophin-glycoprotein complex	[43]
	syndecan-1,2,3 and 4	downregulated during the muscle differentiation	[44]
Nidogen/entactin		supports cross-links between collagen IV and laminin	[45]
Dermatopontin		increases cell adhesion, decreases proliferation, and indorses the myoblast differentiation in C2C12 cells	[31]
Fibronectin		endorses myoblast adhesion and proliferation	[46]
		inhibits differentiation and contributes to fibrillogenesis of collagen	
Laminin		situated in the basal lamina of muscle fibers, promotes integrin expression and activation	[29]
		promotes cell proliferation, adhesion, and differentiation	
Dystrophin and dystroglycan		important links between cytoskeleton and ECM	[29]
		maintain the integrity of cell membrane	
Integrins (α3β1, α6β1, α6β4 and α7β1)		serve as laminin receptors with a high degree of selectivity	[12,47]

### 3.2. Integrin

Integrins are the primary membrane receptors of ECM proteins and enable mechanical interactions between ECM and cells that control myoblast adhesion, proliferation, migration, differentiation, muscle fiber force transmission, and synaptic growth. Integrins are heterodimeric proteins composed of α and β subunits, and in SM, α7β1 is the most common integrin receptor [48,49]. Integrins also enable ‘inside-out’ and ‘outside-in’ signaling between extracellular and intracellular molecules [50].

### 3.3. Decorin and Biglycan

Decorin (DCN) is the most abundant proteoglycan in the perimysium, and its leucine-rich repeats bind to type I collagen around the D- and E-bands [51,52]. DCN has been postulated to be a regulator of type I collagen fibrillogenesis in skeletal muscle because of its binding relationship with type I collagen in vitro [53]. Biglycan has a similar structure to DCN and attaches to type I collagen in the same location as DCN. Collagen fibril diameter in tendon is uneven when DCN or biglycan are absent [54,55]. Biglycan-null mice have a moderate muscular dystrophy phenotype [56], while DCN-null mice’s skin has a lower tensile strength [54], showing the role of these proteoglycans in sustaining normal tissue function.

### 3.4. Dermatopontin

DPT is a tyrosine-rich non-collagenous matrix protein that is mostly expressed in the dermis of the skin and on the surface of collagen fibers [57]. DPT mediates adhesion by binding to cell surface receptors (integrin α3β1), connecting communication between the cell surface of dermal fibroblasts and the ECM environment, increasing transforming growth factor β1 activity, and reducing cell proliferation [58,59,60]. DPT has been shown to promote communication between ECM environments throughout the wound healing process via transforming growth factor β1, DCN, and fibronectin (FN), and it is reported to interact with FN to enhance fibril production and cell adhesion [61]. Recently, we have shown that DPT enhanced cell adhesion, decreased proliferation, and promoted the myoblast differentiation in C2C12 cells [31].

### 3.5. Fibromodulin

FMOD is an ECM protein (belongs to the ECM small-leucine-rich proteoglycan family). It is highly expressed in muscles and connective tissues and was found to be involved in biological regulation processes, such as cell adhesion, and modulation of cytokine activity [62]. FMOD controls the interaction of MSTN with ACVRIIB for myoblast differentiation. FMOD increases the enrollment of MSCs after an injury, which aids in muscle repair, and its upregulation was found to promote the proliferation [15,25]. FMOD was discovered as a MSTN regulator for MSC proliferation and differentiation. FMOD is a component of the ECM that sends out a signal to activate calcium channels via COL1 and ITM2a, causing myogenic differentiation [63]. Considering the above facts FMOD is a crucial factor for the myogenesis by regulating the proliferation and differentiation.

### 3.6. Fibronectin

FN is a glycoprotein present in ECM and found in both soluble and insoluble forms. It helps in several biological process such as organogenesis, hemostasis, cell adhesion and migration [64,65]. There are two types of FN like plasma FN (synthesized by hepatocytes and secreted into blood) and cellular FN (secreted locally by cells) [66,67]. FN assembly has been studied mostly in cell culture and involves FN binding to molecules on cell surfaces, such as syndecans and integrins [68,69]. FN builds a bridge between the cell and its surrounding matrix by binding to collagen and the cell surface. Circulating FN may participate in the formation of ECM in tissues [70]. Syndecans belong to the transmembrane heparan sulfate proteoglycans family and are present in numerous tissues. It was found to be expressed in muscle precursors during embryonic development and in MSC during postnatal life. The role of syndecan was also reported in SM development in flies, turkeys, and mice [71].

### 3.7. Glycosaminoglycan

Glycosaminoglycan (GAG) is a disaccharide linked to polypeptide core to connect two collagen fibers and provide the intermolecular force in COL-GAG matrix [72]. Glycosaminoglycan was found to work as a ground substance in the development of ECM [73]. O-linked glycosaminoglycan was used as an agent for the modification of amino-terminus of DCN, while reduced GAG modification of DCN is a hallmark of gerodermia osteodysplastica [74,75], and reduced DCN glycanation is observed in skin during aging [76]. Overall, it is important for the ECM development and maintenance to avoid aging.

### 3.8. Laminin

Laminin is also an ECM molecule which modulates the activity of MSTN that regulates SM mass [77]. During the embryonic SM development, laminin, concentrated in the limb bud of the myogenic region, participates in the assembly of basal lamina [78]. There are 16 laminin isoforms reported in mammals in the form of recombinant proteins. Ubiquitous laminin 511 and laminin 521 help in the expansion of pluripotent embryonic stem cells [79]. The loss of regenerative potential in laminin-deficient mice, as well as increased satellite cell activity in vivo after supplementing with laminin-111, indicates that this ECM protein plays a key role in the regulation of stem cell function after injury [80].

### 3.9. Dystrophin

Dystrophin is a rod-shaped large protein (430-kDa) molecule. Dystrophin interacts with β-dystroglycan to stabilize dystroglycan and dystrophin glycoprotein complex [81]. The dystrophin glycoprotein complex connects the ECM to the actin cytoskeleton in striated muscle cells and mediates three key functions such as structural stability of plasma membrane, ion homeostasis, and transmembrane signaling [82].

## 4. Co-Culture of Adipose and Muscle Tissue

The development of two different cell kinds in a shared medium is referred to as co-cultures, and the physical contact between cell types may have an effect on cellular function. In biological research, the co-culture system closely resembles in vivo cellular physiology and interactions. Co-culture techniques have been used to explore the relationship between adipose and muscle tissue in a variety of settings, including pharmacological responses, secretory factor production and effect, cell growth, and development [83]. The discovery of adipo-myokine secretory factors, which are generated by adipocytes and myocytes to drive differentiation and proliferation, was made possible by interactions between co-cultured myoblasts and adipocytes [83]. In the co-culture method, differentiation myoblasts control pre-adipocyte differentiation, whereas pre-adipocytes stimulate adipogenic gene expression in MSCs co-cultured with pre-adipocytes. Muscular and adipose tissue are important paracrine and endocrine organs that communicate with one another to control muscle growth and energy homeostasis [84]. Several kinds of muscle interstitial cells, including intramuscular preadipocytes and connective tissue fibroblasts, have been found to interact with MSCs and actively govern postnatal SM development and regeneration. Interstitial adipogenic cells are not only vital for marbling and meat quality, but they also contribute to the MSC niche as a cellular component [85].

## 5. Non-Animal Sourced Scaffolds and the Production of Cultured Meat

Myoblast growth on a scaffold suspended in culture medium inside a bioreactor provides the basis for producing cultured meat. A bioreactor is a device that deceives biological processes through mechanical means. Bioreactors supply nutrients and biomimetic stimuli in a regulated manner to stimulate cell growth, differentiation, and tissue formation [86]. Scaffolds are essential for the development of structured meat products because they provide the structural support required for cells to adhere, divide, and mature. Non-animal derived scaffolds are being explored as potential sources of consistent, chemically defined, low-cost products that avoid/reduce the need for animal slaughter [87], but, at present, no edible scaffolds are commercially available for in vitro meat production [88].

Several companies have started producing cultured meat (available online: https://cellbasedtech.com/lab-grown-meat-companies (accessed on 15 October 2021), and some meat companies are investigating potential scaffolding solutions to reduce their dependencies on animal sources. For example, Matrix Meats (available online: https://www.matrixmeats.com/ (accessed on 19 October 2021)), a company based in the United States, manufactures a non-animal based, edible, nanofiber matrix that mimics natural ECM, and DaNAgreen (available online: http://xn--ok0by47abvffwl.kr/ (accessed on 22 October 2021)), a South Korean company, developed Protinet™-P, an edible scaffold product derived from isolated plant proteins for the manufacture of cell-cultured meat. Another South Korean company, Seawith (available online: http://www.seawith.net/ (accessed on 27 October 2021)), is using algae-based scaffolds to develop its products; algae are nutrient-rich, relatively straightforward, and inexpensive to cultivate.

The meat industry and researchers continue to develop alternatives to natural ECM scaffolds that are cost-effective and biodegradable or edible, but all developed products must also satisfy country-dependent regulatory requirements, and thus, the ingredients used to produce cultivated meat must be readily available, inexpensive, and safe to consume. Polysaccharides such as chitosan, alginate, or cellulose are examples of such ingredients, as are proteins such as zein and complex composites like lignin or textured vegetable protein [4]. Ben–Arye et al. developed a scaffold from textured soy protein, which they described as a cost-effective and novel cell-based meat scaffolding structure. In addition, they demonstrated in a cultured meat prototype that textured soy protein can promote the development of bovine MSCs (Table 2) [89]. It is undesirable to use animal ingredients as scaffolding for cultured meat because the technology’s benefits are only realized when animals are excluded from meat production.

Scaffolds made from salmon gelatin, alginate, agarose, and glycerol show considerable promise as they have relatively large pore sizes (~200 m diameter), are biocompatible, and promote myoblast adhesion (40%) and growth (~24 h duplication time) [88]. Modulevsky et al. showed that 3D cellulose scaffolds made by decellularizing apple hypanthium tissue could be used to culture NIH3T3 fibroblasts, mouse C2C12 muscle myoblasts, and human HeLa cells in vitro [90]. Cellulose, chitin/chitosan, alginate, recombinant silk, PLA (Poly Lactic Acid), and PCL (Poly ε-caprolactone) are examples of non-animal sourced scaffolds that are cheap, consistent, and tunable [87,91]. Polysaccharides are considered attractive biomaterials for tissue engineering applications because of their substantial bioactivities, availabilities, and immunoactivity, and because they are chemically modifiable. Polysaccharides are becoming more widely recognized as potential scaffolds for meat culture due to their biochemical similarities to human ECM. Furthermore, the degradation products of these macromolecules are mostly nontoxic [92].

Decellularization involves the removal of cellular components from tissues using physical and chemical/enzymatic treatments to produce noncellular ECM for therapeutic/scaffolding purposes [93]. In vivo, dense tissues contain complex vascular networks that provide cells with their oxygen and nutrient requirements, but, as yet, no tissue-engineered alternative to this vascularization has been devised for in vitro 3D culture. Decellularized spinach leaves were recently reported to create an edible scaffold with a vascular network that might sustain primary bovine MSCs as they mature into meat that making it a model biomaterial for cultivating meat. Furthermore, spinach leaves are cheap, environmentally sustainable, and edible, and decellularized spinach is free of the animal-derived components present in other biomaterial scaffolds such as gelatin [94].

Some strains of bacteria, such as *Acetobacter* spp., also produce cellulose [95]. Despite having the same cellulosic structure as plant cellulose, bacterial cellulose has higher crystallinity, degrees of polymerization, and water-retaining ability [96], and has been used in emulsified foods to add juiciness and chewiness [97].

**Table 2:** List of non-animal derived microcarrier/ECM components used in scaffold development.

Microcarrier/ECM Components		Source	Edibility	Biodegradability	Cost	Reference
Collagens		recombinant collagen	Yes	Yes	Low	[87]
Gelatin		fish species (salmon)	Yes	NA	Low	[88]
Cellulose scaffold		apple hypanthium (decellularized)	Yes	NA	Low	[90]
Recombinant silk		combined silkworm silk and FN-4RC		Yes	Low	[98,99]
Polysaccharides	Hyaluronic acid	plants	Yes	NA	Low	[92]
	Alginate	0				
	Agar	algae				
Decellularized materials		plant-derived fungal-derived (chitin) decellularized spinach	Yes Yes Yes	Yes	Low	[94]
Bacterial cellulose		bacteria	Yes	Yes	Low	[96,97]

## 6. Recombinant Collagen as a Scaffold for Cultured Meat

Collagen processing systems using mammalian cell-based expression systems for biomedical, cosmetic, or pharmaceutical applications are limited by low yields and high manufacturing costs. To circumvent this problem, researchers have explored systems based on yeasts, insect cells, bacteria, and plants such as tobacco, barley, and corn, as future sources of collagen. Furthermore, animal experiments have shown that collagen can be generated in the mammary glands of transgenic mice and the eggs of transgenic chickens [100,101].

Collagens are attractive materials for the tissue engineering-based assembly of materials for wound healing and drug delivery because of their abilities to function as biological scaffolds [102]. Animal tissues, particularly bovine hides, are the primary source of collagen for most applications [103], though porcine and fish tissues are also viable options. Collagen I is the most common type of collagen used in these applications [104]. Yield enhancement has proven to be a demanding developmental target for recombinant collagen production. Because final product properties are determined by extents of post-translational protein modifications, standardized analogies have not been realized. However, the synthesis of collagen in plants, especially tobacco, is considered an exciting prospect [87].

The use of recombinant variants of natural proteins has resulted in robust production systems that produce pure, biocompatible therapeutics with low immunogenicities on an industrial scale. Recombinant protein technologies are often the only long-term source of therapeutic proteins and can be more cost-effective than isolating proteins from natural sources. As a result, recombinant protein design and development for biomedical applications are critical areas of research. However, the complex structures of collagens, which are biosynthesized intracellularly by collagen-specific chaperones and modifying enzymes, make the development of recombinant collagen variants challenging [101,105]. Recombinant techniques have been used to produce procollagen in bacteria, mammalian cells, insect cell culture, yeast, and plants [87]. Furthermore, recombinant collagen type I is not the only ECM protein that has been developed; recombinant collagens Type II and III, tropoelastin, and fibronectin fragments have also been produced [106,107]. Collagen processing in transgenic tobacco plants, yeast, and/or bacteria may solve some of the problems associated with animal-derived biomaterials. Thus, non-animal derived collagens that closely resemble native collagen offer a possible means toward the production of cultured meat [108].

## 7. Challenges and Future Direction

The cultured meat industry is expanding at a rapid pace because of technological developments aimed primarily at up-scaling production, lowering the cost of growth media, and using non-animal scaffold materials. Despite these efforts, commercial success has been limited by some obvious challenges, which include social and regulatory requirements. In addition, obstacles must be overcome at each developmental step toward the development of cultured meat production processes. Cell culture medium components, particularly serum, growth factors, and ECM (e.g., collagen), should be acquired from different sources while keeping cost and food safety in mind. The biodegradability and edibility of biomaterials should be viewed as priorities when developing 3D scaffolds. Currently, no non-animal derived edible scaffolds are commercially available for cultured meat production, though several plant-based scaffolds are being used on a non-commercial basis.

It is quite difficult to achieve a perfect match to typical meat texture; establishing the circulatory system and connective tissues throughout all three layers of the muscle and fat is considerably more difficult. Co-culture of various cells, such as myoblasts with fibroblasts and adipocytes, can achieve a comparable matching, however the concern is that each cell line is distinct and requires its own growth and differentiation conditions. Ignoring these factors leads to sub-optimal development, which has a direct impact on the texture and flavor of the in vitro meat [109].

Furthermore, intelligent, high-efficiency bioreactors are required for the scale-up of cultured meat production, and this is one of the greatest challenges that the cultured meat industry has yet to overcome. Finally, the cost and food safety concerns of each component in the manufacturing process must be rigorously addressed.

## Data Availability

Not Applicable.
